# Plant cell wall proteomics: the leadership of *Arabidopsis thaliana*

**DOI:** 10.3389/fpls.2013.00111

**Published:** 2013-05-01

**Authors:** Cécile Albenne, Hervé Canut, Elisabeth Jamet

**Affiliations:** ^1^Laboratoire de Recherche en Sciences Végétales, Université de Toulouse, UPS, UMR 5546Castanet-Tolosan, France; ^2^CNRS, UMR 5546Castanet-Tolosan, France

**Keywords:** *Arabidopsis thaliana*, cell wall, mass spectrometry, peptidomics, proteomics

## Abstract

Plant cell wall proteins (CWPs) progressively emerged as crucial components of cell walls although present in minor amounts. Cell wall polysaccharides such as pectins, hemicelluloses, and cellulose represent more than 90% of primary cell wall mass, whereas hemicelluloses, cellulose, and lignins are the main components of lignified secondary walls. All these polymers provide mechanical properties to cell walls, participate in cell shape and prevent water loss in aerial organs. However, cell walls need to be modified and customized during plant development and in response to environmental cues, thus contributing to plant adaptation. CWPs play essential roles in all these physiological processes and particularly in the dynamics of cell walls, which requires organization and rearrangements of polysaccharides as well as cell-to-cell communication. In the last 10 years, plant cell wall proteomics has greatly contributed to a wider knowledge of CWPs. This update will deal with (i) a survey of plant cell wall proteomics studies with a focus on *Arabidopsis thaliana*; (ii) the main protein families identified and the still missing peptides; (iii) the persistent issue of the non-canonical CWPs; (iv) the present challenges to overcome technological bottlenecks; and (v) the perspectives beyond cell wall proteomics to understand CWP functions.

## Introduction

**Figure 1:**
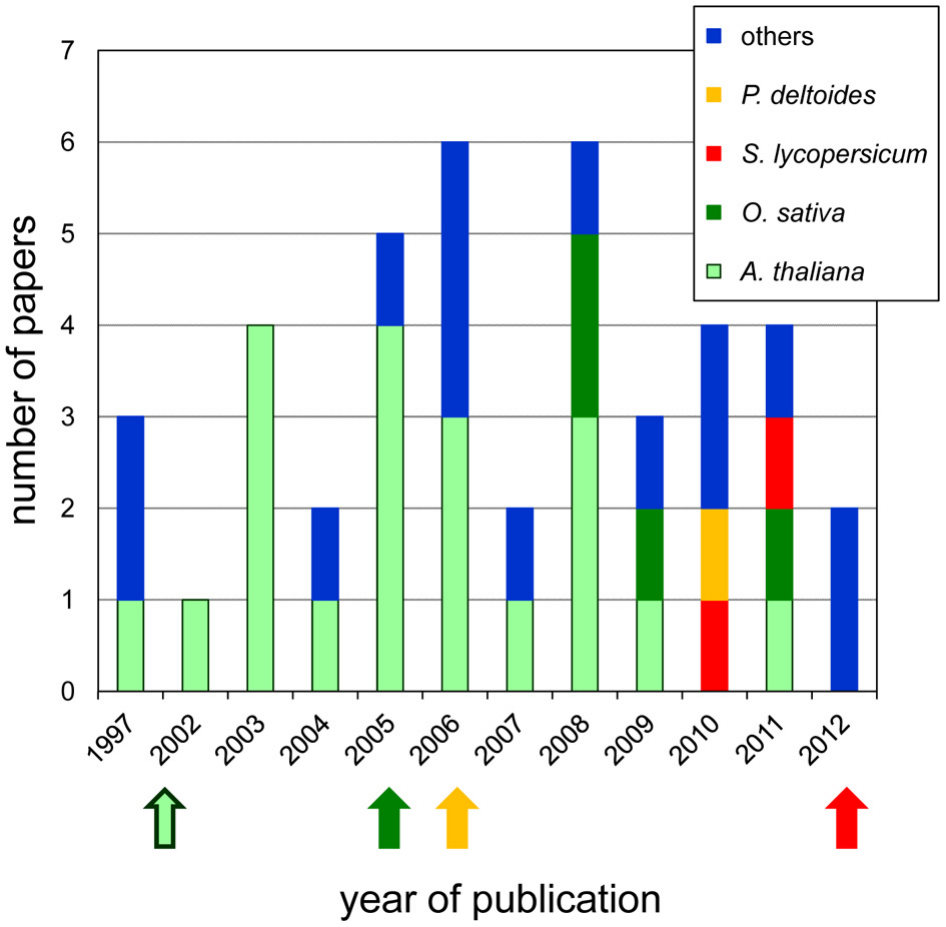
**Occurrence of cell wall proteomics papers since 1997.** The number of papers published each year is represented with colored bars for each plant: *A. thaliana* (green), *O. sativa* (pink), *P. deltoides* (yellow), *S. lycopersicum* (red), and others (blue). The arrows show the year of the genomic sequence release for each of them. Note that the genomic sequence of *P. trichocarpa* was used for protein identification in *P. deltoides*.

Plant primary cell walls are mainly composed of polysaccharide networks such as cellulose microfibrills, hemicelluloses wrapping and interlacing cellulose microfibrills and pectins (Carpita and Gibeaut, 1993). After the end of cell growth, secondary walls which contain additional compounds such as lignins, wax or cutin, are synthesized. Cell wall proteins (CWPs) play critical roles in plant cell walls during development and adaptation to environmental cues (Fry, 2004; Passardi et al., 2004). For this reason, extensive studies leading to their identification and characterization have been undertaken. Cell wall proteomics started about 10 years ago when the first plant genome sequences became available. Nowadays, there are about 40 papers covering this field (Figure 1), half of them concerning *Arabidopsis thaliana* whose genome was available in 2000 (Arabidopsis Genome Initiative, 2000). The availability of new genome sequences such as those of *Oryza sativa* (International Rice Genome Sequencing Project., 2005), *Populus trichocarpa* (Tuskan et al., 2006) and *Solanum lycopersicum* (Tomato Genome Consortium, 2012) enlarged the range of plant proteomics studies.

For plant cell wall proteomics studies, organs or cell suspension cultures have been used as starting materials containing cells surrounded by primary and/or secondary walls. Various experimental approaches were undertaken to characterize cell wall proteomes. Five specific features of CWPs need to be emphasized to understand them. (i) CWPs represent only 5–10% of the cell wall mass (Cassab and Varner, 1988). They are embedded in a complex matrix of carbohydrate polymers, aromatic compounds, wax or cutin depending on the type of cell walls. (ii) CWPs may interact with cell wall components by non-covalent linkages (Carpin et al., 2001; Spadoni et al., 2006). They can also be covalently linked, thus forming insoluble networks, like structural proteins networks of Proline-Rich Proteins (PRPs) or extensins (Brisson et al., 1994; Brady et al., 1996). (iii) Contrary to other sub-cellular compartments, plant cell walls constitute an open space connecting the cells in a tissue. It is located between the cell plasma membrane and the cuticle in aerial organs or the suberin layer in roots conferring to the plant surface waterproof qualities and protection against biotic and abiotic stresses (Thomas et al., 2007; Javelle et al., 2010). (iv) Most CWPs are basic proteins (Jamet et al., 2008). (v) Most CWPs undergo post-translational modifications (PTMs), like hydroxylation of proline (Pro) residues converting them to hydroxyproline (Hyp), *N*-glycosylation, *O*-glycosylation or addition of a glycosylphosphatidylinositol (GPI)-anchor (Kieliszewski and Lamport, 1994; Spiro, 2002; Faye et al., 2005).

For each step of the cell wall proteomics flowchart, the specificities of CWPs must be taken into account: plant fractionation, protein extraction, protein separation, protein identification by mass spectrometry (MS), and bioinformatics. Indeed, CWPs can be tightly trapped into the extracellular matrix and escape the extraction procedure. They may not be resolved at the step of separation by two-dimensional electrophoresis (2D-E) because they are mainly basic glycoproteins (Jamet et al., 2008). Finally, the databases used for protein identification using MS data contain no information about PTMs such as glycosylation, thus preventing the identification of some of them.

In this review, we will give a survey of plant cell wall proteomics studies with a focus on *A. thaliana* because this plant provides the best documented cell wall proteomes. The main protein families identified and the persistent issue of the non-canonical CWPs will be discussed. Finally, we will provide perspectives in the field of plant cell wall proteomics, going beyond the present data with systems biology approaches and peptidomics to decipher the roles of proteins and peptides in cell walls.

## Materials and methods

All the *A. thaliana* proteins reported in this review have been analyzed with different bioinformatics software to predict their sub-cellular localization and their functional domains using *ProtAnnDB* (http://www.polebio.lrsv.ups-tlse.fr/ProtAnnDB/index.php) as previously described (San Clemente et al., 2009). Briefly, the following programs have been used for prediction of sub-cellular localization: TargetP (http://www.cbs.dtu.dk/services/TargetP/), SignalP (http://www.cbs.dtu.dk/services/SignalP/), Predotar (http://urgi.versailles.inra.fr/predotar/predotar.html), Aramemnon (http://aramemnon.botanik.uni-koeln.de/) and TMHMM (http://www.cbs.dtu.dk/services/TMHMM-2.0/). The programs used for prediction of functional domains were PROSITE (http://prosite.expasy.org/), Pfam (http://pfam.sanger.ac.uk/), and InterPro (http://www.ebi.ac.uk/interpro/).

## A survey of plant cell wall proteomics

### Extracellular proteomes

In this review, we will focus on different type of extracellular proteomes, commonly named as call wall proteomes. For example, secretome, in which all of the secreted proteins of a cell suspension culture, roots or seedling are collected in liquid culture media. Another type of extracellular proteome encompasses apoplastic proteomes in which proteins from the cell wall can be eluted by vacuum infiltration with various solutions. Extraction of proteins from purified cell walls with various solutions is the third category of cell wall proteome that have been used to elute loosely bound CWPs. In addition, sub-proteomes such as *N*-glycoproteomes and a GPI-anchored proteome have been analyzed. All the extracellular proteomes have been obtained with different plants like *A. thaliana* (see Table 1), *Cicer arietinum* (Bhushan et al., 2006, 2011), *Glycine max* (Komatsu et al., 2010), *Helianthus annuus* (Pinedo et al., 2012), *O. sativa* (Chen et al., 2008a; Jung et al., 2008; Cho et al., 2009; Zhou et al., 2011), *Medicago sativa* (Watson et al., 2004; Verdonk et al., 2012), *Nicotiana benthamiana* (Goulet et al., 2010), *Nicotiana tabacum* (Robertson et al., 1997; Dani et al., 2005; Morel et al., 2006; Delannoy et al., 2008; Millar et al., 2009), *Populus deltoides* (Pechanova et al., 2010), *S. lycopersicum* (Robertson et al., 1997; Yeats et al., 2010; Catalá et al., 2011), *Solanum tuberosum* (Fernández et al., 2012; Lim et al., 2012) and *Zea mays* (Zhu et al., 2006, 2007). Besides, several xylem sap proteomes have been analyzed and were found to be very close to cell wall proteomes (Kehr et al., 2005; Alvarez et al., 2006; Dafoe and Constabel, 2009; Ligat et al., 2011). With 20 published papers (Table 1) and 500 proteins with predicted signal peptide identified, the most studied plant is *A. thaliana*. Its genome was the first one to be sequenced, thus allowing a precise identification of the proteins. Altogether, between one fourth and one third of the expected cell wall proteome of *A. thaliana* has been identified (Jamet et al., 2006). The second most studied plant is *O. sativa* with 270 proteins with predicted signal peptide identified. When no genome information is available, protein identification relies on the availability of expressed sequenced tags (ESTs) or cDNAs (Lim et al., 2012). Alternatively, proteins are identified by sequence homology. In this case, it is not possible to obtain precise identification of proteins and to distinguish between members of multigene families like in *C. arietinum* or *H. annuus* (Bhushan et al., 2006; Pinedo et al., 2012).

**Table 1:** **Cell wall proteomes of *A. thaliana***.

**Organ**	**References**	**Type of proteome**	**Total number of identified proteins (% without predicted signal peptide)^c^**	**Number of proteins with predicted signal peptide^c^**
Culture medium of roots	Basu et al., 2006	Secretome	52 (31)	36
Stems	Minic et al., 2007	*N*-glycoproteome	102 (12)	90
Leaves	Haslam et al., 2003	Apoplastic proteome	10 (10)	9
Leaves	Boudart et al., 2005	Apoplastic proteome	93 (7)	87
Etiolated hypocotyls (5 days)	Feiz et al., 2006	Cell wall proteome	96 (13)	84
Etiolated hypocotyls (5 and 11 days)	Irshad et al., 2008	Cell wall proteome	173 (21)	137
Etiolated hypocotyls (11 days)	Zhang et al., 2011	Cell wall glycoproteome	129 (2)	127
Seedlings (10 days) (oligogalacturonides)	Casasoli et al., 2008^a^	Apoplastic proteome	20 (30)	14
Cell suspension cultures (3 days)	Bayer et al., 2006	Cell wall proteome	792 (87)	106
Cell suspension cultures (5 days)	Kwon et al., 2005	Cell wall proteome^b^	39 (13)	34
Cell suspension cultures (5 days)	Chivasa et al., 2002	Cell wall proteome	72 (64)	26
Cell suspension cultures (5 days)	Robertson et al., 1997	Cell wall proteome^b^	33 (46)	18
Cell suspension cultures (7 days)	Borderies et al., 2003	Cell wall proteome^b^	95 (48)	50
Culture medium of calli (21 days)	Borner et al., 2003	GPI-anchored proteome	30 (0)	30
Culture medium of cell suspension cultures (4 days)	Oh et al., 2005	Secretome	45 (47)	24
Cell suspension cultures (7 days) (fungal elicitors)	Ndimba et al., 2003^a^	Cell wall proteome	6 (0)	6
Culture medium of cell suspension cultures (7 days) (salicylic acid treatment)	Cheng et al., 2009	Secretome	74 (47)	39
Culture medium of cell suspension cultures (7 days) (phosphate deficiency)	Tran and Plaxton, 2008^a^	Secretome	37 (43)	21
Protoplasts (1 h)	Kwon et al., 2005	Cell wall proteome^b^	71 (65)	25
Protoplasts (3 h)	Kwon et al., 2005	Cell wall proteome^b^	66 (65)	23
Liquid cultured etiolated seedlings	Schultz et al., 2004	AGP proteome	12 (0)	12
Culture medium of etiolated seedlings	Charmont et al., 2005	Secretome	49 (10)	44

a *Protein identification has been only performed on protein spots showing variation between control and treated samples*.

b *These proteomes have been obtained by washing of cells or protoplasts with various salt solutions (see Table A1)*.

c *All the bioinformatic predictions of sub-cellular localization have been done as described in Materials and Methods to allow the comparison between the A. thaliana cell wall proteomes*.

### Strategies in plant cell wall proteomics

**Figure 2:**
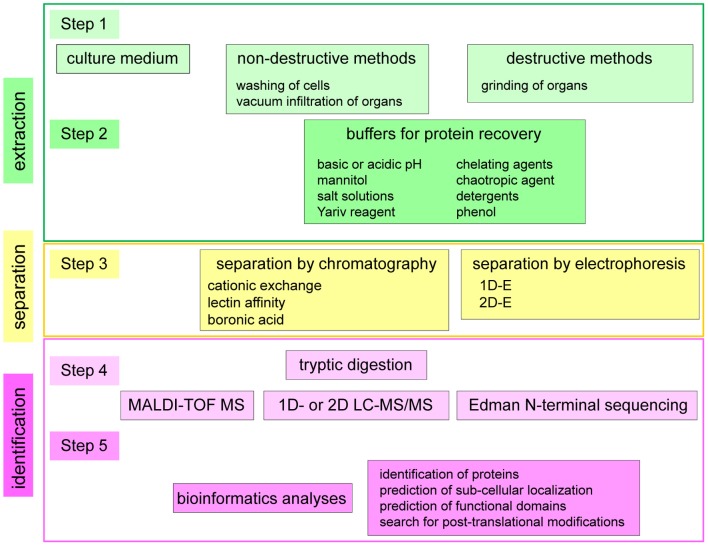
**Synopsis of the different strategies used for the study of cell wall proteomes and secretomes.** Five main steps have been identified in the published strategies and different combinations of the five steps have been used. Steps 1 and 2 lead to protein extraction. Step 3 consists in protein separation. Steps 4 and 5 lead to protein identification by combining MS or Edman N-terminal sequencing and bioinformatics. In some cases, one step is performed twice or even several times, e.g., steps 2 and 3, by modifying the composition of the buffer used for protein recovery or doing two subsequent steps of protein separation. In other cases, one step can be skipped, like step 2 for the analysis of secreted proteins present in culture medium, or step 3 when proteins are directly analyzed by MS.

Many different strategies have been used to identify extracellular proteins of plants. A synopsis of the different experimental procedures is presented in Figure 2 and Table A1 in five general steps. Steps 1 and 2 lead to protein extraction. Step 3 consists in protein separation. Steps 4 and 5 lead to protein identification. The first step distinguishes: (i) studies of secretomes in which only proteins spontaneously released in culture media are analyzed (Charmont et al., 2005; Oh et al., 2005; Basu et al., 2006; Tran and Plaxton, 2008; Cheng et al., 2009); (ii) the release of proteins by non-destructive methods in which the integrity of the cell plasma membranes is preserved either by vacuum infiltration of tissues (Haslam et al., 2003; Boudart et al., 2005; Casasoli et al., 2008) or by washing of cells cultured in liquid medium (Robertson et al., 1997; Borderies et al., 2003; Kwon et al., 2005); and (iii) the release of proteins by destructive methods starting with a grinding of the tissues, thus mixing intracellular and extracellular compartments. In this case, either cell walls were purified prior to protein extraction (Chivasa et al., 2002; Ndimba et al., 2003; Feiz et al., 2006; Minic et al., 2007; Irshad et al., 2008; Zhang et al., 2011) or the tissues were ground prior to isolation of *N*-glycosylated proteins by lectin affinity chromatography (Minic et al., 2007). In the case of the GPI-anchored proteome, the first step consisted in the preparation of a membrane fraction followed by the cleavage of GPI-anchors by phosphatidylinositol-specific phospholipase C (Pi-PLC) (Borner et al., 2003).

The second step (Figure 2) is very diverse using different solutions to extract proteins. These solutions can be acidic or basic (Feiz et al., 2006; Casasoli et al., 2008). Their main components are: salts (NaCl, CaCl_2_, MgCl_2_, KCl, or LiCl) or osmotic agents (mannitol) (Borderies et al., 2003; Boudart et al., 2005; Kwon et al., 2005; Feiz et al., 2006); chelating agents (EDTA or CDTA) (Robertson et al., 1997; Boudart et al., 2005); detergents (SDS, Triton or CHAPS) (Chivasa et al., 2002; Borner et al., 2003); phenol (Bayer et al., 2006); and/or chaotropic agents (urea and thiourea) (Chivasa et al., 2002). The β-glucosyl Yariv reagent has been used to isolate arabinogalactan proteins (AGPs) (Schultz et al., 2004). In some cases, several salt solutions have been used successively (Chivasa et al., 2002; Borderies et al., 2003; Boudart et al., 2005; Feiz et al., 2006; Irshad et al., 2008; Zhang et al., 2011). As mentioned above, in the case of the GPI-anchored proteome, a Pi-PLC has been used (Borner et al., 2003). The methods used to extract CWPs have been previously described in detail (Feiz et al., 2006; Jamet et al., 2008).

The third possible step is protein separation (Figure 2). It can be done by chromatography (cationic exchange, lectin affinity, boronic acid), and/or by 1D- or 2D-E. Cationic exchange chromatography has been performed under physico-chemical conditions similar to those found in cell walls, that is an acidic medium at a pH around 4.5 at which basic proteins are positively charged (Boudart et al., 2005; Irshad et al., 2008). Affinity chromatography on Concanavalin A (ConA) has been artfully used to isolate *N*-glycoproteins from a total extract of proteins (Minic et al., 2007). As expected, most of the identified proteins were predicted to be addressed to the secretion pathway where *N*-glycosylation occurs. Other lectins have been used to separate proteins extracted from cell walls: *Artocarpus integrifolia* Lectin (AIL) specific for α-Gal residues, PeaNut Agglutinin (PNA) specific for β-Gal residues, and wheat germ agglutinin (WGA) specific for GlcNAc residues (Zhang et al., 2011). With regard to separation of proteins by electrophoresis, 2D-E has shown limitations due to the fact that CWPs are mainly basic glycoproteins (Jamet et al., 2008). Considering the number of identified proteins, the most efficient cell wall proteomics analyses have been performed using two steps of protein separation (Boudart et al., 2005; Minic et al., 2007; Irshad et al., 2008; Zhang et al., 2011).

Two additional steps are necessary to achieve protein identification (steps 4 and 5, Figure 2). The fourth step consists in proteolytic digestion of proteins and MS analyses of peptides, using Matrix-Assisted Laser Desorption Ionization-Time Of Flight (MALDI-TOF) MS (Boudart et al., 2005; Kwon et al., 2005; Irshad et al., 2008), liquid chromatography (LC)-MS/MS (Minic et al., 2007; Casasoli et al., 2008; Zhang et al., 2011) or 2D-LC-MS/MS (Basu et al., 2006; Bayer et al., 2006; Cheng et al., 2009). Trypsin is the most widely used protease. In a few cases, Edman N-terminal sequencing has been performed (Robertson et al., 1997; Schultz et al., 2004). When proteins are heavily glycosylated, like *O*-glycoproteins, it is necessary to deglycosylate them with hydrogen fluoride (HF) to get access to their polypeptide skeleton (Schultz et al., 2004). The fifth step consists in bioinformatics analyses to identify proteins, predict their sub-cellular localization, their functional domains and eventually get information about their PTMs (San Clemente et al., 2009).

Different complementary strategies are now available to study plant cell wall proteomes. It is possible to design the most relevant flowchart for a new cell wall proteomics study and to perform it in an efficient way. The main limitation remains the availability of genomic sequences for many plants of agronomic interest.

### Plant cell wall glycoproteomes

During their secretion, proteins undergo glycosylation which is one of the most common and complex PTM known to control many physiological processes (Faye et al., 2005). Glycosylation is of two main types, namely *N*- and *O*-glycosylation, depending on the nature of the amino acid bearing them. Unlike yeast and mammalian glycoproteins which are extensively studied, plant glycoproteins are still poorly characterized. Hyp-rich glycoproteins (HRGPs) undoubtedly constitute the most documented plant cell wall *O*-glycoprotein superfamily (Kieliszewski, 2001; Tan et al., 2012; Velasquez et al., 2012). A few *N*-glycoproteins have been studied in detail, e.g., a peroxidase (Lige et al., 2001), an α-mannosidase (Kimura et al., 1999) or a polygalacturonase inhibiting protein (PGIP) (Lim et al., 2009) for which glycosylation has been shown to contribute to activity. Beyond the study on targeted glycoproteins, the concept of glycoproteomics is now emerging in plants. New analytical pipelines are available (Song et al., 2011; Ruiz-May et al., 2012). They aim at detection, enrichment and MS analysis of large sets of glycoproteins.

A few systematic surveys have been carried out so far on plants. Minic et al. were the first ones to use a ConA lectin chromatography step to capture *N*-glycoproteins from a protein extract of *A. thaliana* (Minic et al., 2007). A similar approach has been developed to characterize the *N*-glycoproteome of *S. lycopersicum* (Catalá et al., 2011). Finally, Zhang et al. enlarged the coverage of the *A. thaliana* cell wall glycoproteome using multi-dimensional lectin chromatography and boronic acid chromatography (Zhang et al., 2011). The obtained sub-proteomes mostly corresponded to *N*-glycoproteomes with only few *O*-glycoproteins detected. Plant glycoproteomics is only at its premise and is undoubtedly a very promising approach toward an integrated study of both sugar and proteins moieties to gain new insight into the structure and function of glycoproteins.

## Main CWP families

### Grouping of CWPs: principles and drawbacks

A major challenge is to interpret cell wall proteomics data, in other words, to get a biological message from a list of proteins. In a first effort, it is tempting to group proteins to get an overview of the extracellular proteome, to highlight specific proteins or protein families in the physiological context of interest, or to identify house-keeping proteins. This is a difficult exercise because most of the identified proteins have no experimentally defined function. Another difficulty is that two series of proteins can be distinguished in all cell wall proteomes: those having a signal peptide predicted with at least two bioinformatics programs, and those having no predicted signal peptide or having a motif that addresses them to an intracellular compartment. Only those having a *bona fide* predicted signal peptide are named CWPs in this review. This point will be discussed below.

From the fifth step of the proteomics flowchart (Figure 2), bioinformatics analyses lead to group proteins in families either by sequence comparison to proteins present in databases and already annotated, or by search for functional domains as defined in domain repertoires like PROSITE, Pfam, or InterProScan. Two types of classification have then been proposed. Both of them have drawbacks and suffer from ambiguity. The first type is based on the physiological processes in which the proteins are assumed to be involved, like growth and development, stress or defense against pathogens. The drawback is that it can be difficult to sort the proteins. For example, glycoside hydrolases (GHs) could be involved in both plant development and defense (Kasprzewska, 2003). In the same way, proteases could be involved in protein turnover or in signaling by releasing biologically active peptides (Berger and Altmann, 2000; Hunt et al., 2010; Leasure and He, 2012). The second type of classification is based on predicted functional domains and possible partners or targets (i.e., polysaccharides, lipids or proteins) of CWPs in cell walls. The drawbacks are the followings: (i) all the proteins do not have a predicted biochemical activity; (ii) it is difficult to assign proteins with several functional domains to a class as exemplified below. Of course, all these classifications need to evolve to take into account new experimental results demonstrating protein functions.

### An example of functional classification of CWPs

In this review, we present a functional classification taking into account predicted functional domains as well as possible partners or targets in cell walls. *A. thaliana* CWPs are taken as an example. Nine functional classes listed in Table 2 have been proposed (Jamet et al., 2008). The most populated functional class is that of proteins acting on carbohydrates. It represents about one fourth of the proteomes (25.6%) and it includes GHs, carbohydrate esterases (CEs), polysaccharide lyases (PLs) and expansins. The importance of such proteins is not surprising since polysaccharides constitute the largest fraction of cell walls and are constantly submitted to remodeling during plant development or in response to environmental cues (Fry, 2004; Cosgrove, 2005). The second most predominant class of CWPs is that of oxido-reductases (14.6%), like peroxidases, multicopper oxidases, blue copper binding proteins, and berberine bridge enzymes. Again, the importance of this class was expected because many oxidation reactions occur in the extracellular matrix to modify polymer networks involving carbohydrates, aromatic compounds, or structural proteins (Passardi et al., 2004). However, the biochemical functions of proteins homologous to berberine bridge enzymes and of blue copper binding proteins in cell walls are not known (Nersissian and Shipp, 2002). Then, numerous proteases have been found in cell wall proteomes (11.2%). Until recently, the roles of such enzymes have probably been under-estimated in cell walls. They could be involved in protein turnover, protein maturation or release of biologically active peptides (Van Der Hoorn, 2008). Nothing is known about CWP turnover. The maturation of enzymes having N- or C-terminal pro-peptides or N-terminal inhibitory domains has been demonstrated only in a few cases, such as type I pectin methylesterases (PMEs) (Wolf et al., 2009) and some GHs (Lee et al., 2003; Minic et al., 2004; Albenne et al., 2009). It was also shown that the AtSBT1.7 Ser protease plays a role in mucilage release from *A. thaliana* seed coat (Rautengarten et al., 2008). It is assumed that this protease contributes to the degradation or the maturation of cell wall modifying enzymes. The class of CWPs possibly involved in signaling (6.6%) is a difficult one. It comprises proteins like AGPs and proteins with transmembrane domains which are predicted to be plasma membrane receptors having extracellular domains. The roles of AGPs are not completely understood. Besides their roles in signaling (Seifert and Blaukopf, 2010), AGPs could also contribute to cell wall mechanical properties (Seifert and Roberts, 2007). The identification of receptors mostly relies on peptides located in the extracellular domain, but they do not really belong to cell wall proteomes. Finally a protease like stomatal density and distribution 1 (SDD1) could also be included in the class of signaling proteins because it is assumed to generate a extracellular signal to control the stomatal pattern (Von Groll et al., 2002). The next class of CWPs is that of proteins predicted to be related to lipid metabolism which is unexpectedly populated (5.8%). Such proteins are assumed to be involved in cuticle formation. However, the cuticle does not represent a major part of the organs analyzed. Other roles might be possible for these proteins. For example, a lipid transfer protein (LTP) has been assumed to be involved in cell wall extension by interacting with the cellulose/xyloglucan network of tobacco cell walls (Nieuwland et al., 2005). The class of structural proteins (1.6%) only groups a few proteins like Glycine-Rich Proteins (GRPs), PRPs, and Leucine-Rich Repeat Extensins (LRXs). The problem of CWP classification appears again with LRXs which could also be involved in signaling or be classified among proteins having predicted interaction domains (Baumberger et al., 2001; Leiber et al., 2010). No extensin has been identified in the published cell wall proteomes probably because they are covalently cross-linked (Wilson and Fry, 1986). The strategies presently used for cell wall proteomics fail to efficiently isolate such proteins. The class of CWPs with interaction domains (11.0%) presently groups proteins having predicted carbohydrate binding domains, Leucine-Rich-Repeat (LRR) domains assumed to be involved in protein–protein interactions and enzyme inhibitors. This class can be split according to these three categories of CWPs (Catalá et al., 2011). A better knowledge of the function of proteins interacting with polysaccharides will also contribute to a more precise classification. The group of miscellaneous proteins (11.0%) is the Achilles'heel of the classification since it comprises all the proteins which cannot be put elsewhere. A few protein families emerge from this group like purple acid phosphatases (Wang et al., 2011), phosphate-induced proteins (Farrar et al., 2003) and germins (Membré et al., 2000). Finally, about one eighth of the cell wall proteomes correspond to proteins of yet unknown function with no predicted functional domain or a predicted domain of unknown function (DUF). This is a puzzling class of proteins which will probably reveal new functions in cell walls. It is expected to disappear when these proteins are characterized.

**Table 2:** **A classification of *A. thaliana* CWPs in 9 functional classes according to their predicted functional domains and their possible partners in cell walls**.

**Functional class**	**Protein families**	**Possible partners in cell walls**	**% *A. thaliana* proteomes**
Proteins acting on carbohydrates			25.7
	Glycoside hydrolases (GHs)	Carbohydrates	19.4
	Carbohydrate esterases (CEs)	Pectins	2.4
	Polysaccharide lyases (PLs)	Pectins	0.6
	Expansins	Cellulose/hemicellulose	2.4
Oxido-reductases			14.6
	Peroxidases	Carbohydrates/Structural proteins/Lignins	6.2
	Multicopper oxidases		2.0
	Blue copper binding proteins		2.2
	Berberine bridge enzymes		2.2
Proteases			11.2
	Asp proteases	Proteins	2.8
	Cys proteases	Proteins	1.6
	Ser proteases	Proteins	3.6
	Ser carboxypeptidases	Proteins	2.2
Proteins with interaction domains			11.0
	Lectins	Carbohydrates	2.4
	Leucine-rich-repeat domains (LRRs)	Proteins	2.6
	Enzyme inhibitors	Enzymatic proteins	4.0
Proteins possibly involved in signaling			6.6
	Arabinogalactan proteins (AGPs)		2.4
	Fasciclin-AGPs (FLAs)		1.2
	Receptors		2.4
Structural proteins			1.6
	Glycine-rich proteins (GRPs)		0.2
	Leucine-rich-repeat extensins (LRXs)	Proteins	0.8
	Proline-rich proteins (PRPs)	Proteins	0.6
Proteins related to lipid metabolism			5.8
	Lipase GDSL	Lipids	2.4
	Lipid transfer proteins (LTPs)	Lipids	1.8
Miscellaneous proteins			11.0
	Purple acid phosphatases (PAPs)		1.2
	Phosphate-induced (phi) proteins		1.0
	Germin		1.6
Unknown function			12.5
	Domains of unknown function (DUFs)		5.0
	No clue		4.4

*The annotation of proteins is based on the presence of functional domains as defined in the PROSITE, Pfam, and InterPro bioinformatics programs. Only the major protein families are mentioned in each functional class*.

### *WallProtDB*, a cell wall proteomics database

The classification of proteins described above has been used to build up the *WallProtDB* database (http://www.polebio.scsv.ups-tlse.fr/WallProtDB/) (Pont-Lezica et al., 2010; Ligat et al., 2011). The 20 published *A. thaliana* cell wall proteomes listed in Table 1 (described in more details in Table A1) have been subjected to the same bioinformatics software pipeline (*ProtAnnDB*) in order to compare them more accurately. The number of CWPs identified in these proteomes is very variable, ranging from 6 to 137. The less populated proteomes are a leaf apoplast proteome (Haslam et al., 2003), the AGP proteome (Schultz et al., 2004) and those focused on proteins which level of accumulation changes in response to a treatment (Ndimba et al., 2003; Casasoli et al., 2008; Tran and Plaxton, 2008). On the contrary, the most populated proteomes are those relying on efficient CWP extraction (Boudart et al., 2005) or separation (Minic et al., 2007; Irshad et al., 2008; Zhang et al., 2011), or on the most sensitive MS techniques (Bayer et al., 2006). In addition to the *A. thaliana* cell wall proteomes (500 CWPs), the cell wall proteomes of *O. sativa* and a *B. oleracea* xylem sap proteome have been included in *WallProtDB*, thus representing about 1000 CWPs.

## The case of non-canonical CWPs

Apart from the proteins having predicted signal peptides, all the cell wall proteomes contain proteins which are not predicted to be secreted and proteins predicted to be endoplasmic reticulum resident proteins. The proportion of these non-canonical CWPs varies from none in the case of the AGP and GPI-anchored proteomes (Borner et al., 2003; Schultz et al., 2004) to 87% (Bayer et al., 2006), the average being 30% (Table 1). The cell wall proteomes containing the lowest proportion of non-canonical CWPs have been obtained with the following strategies: secretome analysis (Charmont et al., 2005), extraction of apoplastic fluids with salt solutions (Boudart et al., 2005), affinity chromatography on lectins to isolate glycoproteins (Minic et al., 2007; Catalá et al., 2011; Zhang et al., 2011) and cell wall purification with an adapted protocol followed by extraction of proteins with salt solutions (Feiz et al., 2006). Apart from the limitations of bioinformatics programs to predict sub-cellular localization (Imai and Nakai, 2010), the difficulties mentioned above to preserve membrane integrity or to purify cell walls have to be taken into account to understand these contrasting results. Moreover, the facts that the percentage of non-canonical CWPs varies between experiments and that these proteins are not always the same, indicate that most of them are probably intracellular contaminants. However, it cannot be excluded that some of them are present in cell walls. This point has been recently reviewed (Rose and Lee, 2010).

Several authors used prediction of sub-cellular localization with SecretomeP which performs *ab initio* predictions of non-classical, i.e., not signal peptide-triggered, protein secretion for mammalian proteins (http://www.cbs.dtu.dk/services/SecretomeP/) (Bendtsen et al., 2004). However, this software is not well-adapted to plant proteins since it has been designed for mammalian proteins. Moreover, only a small proportion of the non-canonical proteins identified in cell wall proteomics studies gave a score above threshold (Jamet et al., 2008; Pechanova et al., 2010; Bhushan et al., 2011; Fernández et al., 2012).

Unfortunately, experimental data are too scarce to confirm the localization of all the non-canonical CWPs. In animal cells, several alternative mechanisms of protein secretion have been proposed and partly demonstrated (Nickel and Seedorf, 2008). Unconventional secretory proteins seem to share several common features like (i) no leader sequence, (ii) absence of PTMs specific for ER or Golgi apparatus, and (iii) secretion not affected by brefeldin A which blocks the classical ER/Golgi-dependent secretion pathway. A jacalin-related lectin has been first identified in extracellular fluids of sunflower seedlings and then demonstrated to be extracellular by immunolocalization (Pinedo et al., 2012). This is the only case where the three criteria defined for animal unconventional secretory proteins described above were met, leading to the assumption of the release of exosomes to the extracellular matrix (Regente et al., 2012). In addition, a few moonlighting proteins were described like a rice α-amylase (GH13 family) which was shown to be present in both cell walls and plastids (Chen et al., 2004).

There is an urgent need for systematic localization of plant proteins by (i) biochemical strategies like isotope tagging successfully used for the membrane organelle proteome of *A. thaliana* (Dunkley et al., 2006), (ii) immunolocalization to get a reliable protein atlas as done in the Human Protein Atlas project (Lundberg and Uhlén, 2010), (iii) green fluorescent protein (GFP) tagging (Heazlewood et al., 2005) or (iv) a yeast secretion trap assay (Lee and Rose, 2012). An interesting tool is SUBA3 (SUBcellular localization database for Arabidopsis proteins) which collects bioinformatics and experimental data of sub-cellular localization (http://suba.plantenergy.uwa.edu.au/) (Heazlewood et al., 2007). The precise identification of secreted proteins devoid of predicted signal peptide will allow the demonstration of the existence of alternative secretion pathways in plant cells and the design of bioinformatics software able to predict non-classical secretion of plant proteins.

## Present challenges: overcoming technological bottlenecks

Plant cell wall proteomics has been a very active research area during the last 10 years, and is rapidly expanding with the availability of new genome sequences. However, knowledge on plant CWPs will gain new insight thanks to new methodological and technological developments aiming at the identification of low-abundant proteins, the characterization of protein–protein complexes and the description of PTMs.

### Toward complete cell wall proteomes

Proteomics studies aim at providing a global description of proteins present in a biological extract. However, the complexity of protein samples renders difficult their exhaustive analysis since (i) a few highly-abundant proteins can mask low-abundant proteins, and (ii) the dynamic range of proteins can be very broad, i.e., up to 12 orders of magnitude (Corthals et al., 2000). To overcome these limitations, new separation techniques have been developed, namely depletion and equalization methods. These methods have first proven their efficiency for mammalian and microbial systems and are now emerging for plants. Plant depletion methods described so far mostly concern the depletion of storage proteins or ribulose-1,5-biphosphate carboxylase/oxygenase (RUBISCO). A fast and simple fractionation technique to precipitate legume seed storage proteins has been developed, allowing the detection of 541 low-abundant proteins of *G. max* seeds after a 2D-E separation (Krishnan et al., 2009). A similar approach has been carried out to precipitate RUBISCO from soybean leaf soluble protein extract, permitting the detection of 230 new protein spots (Krishnan and Natarajan, 2009). Another RUBISCO depletion method based on immunocapture has been successfully performed to detect low-abundant proteins differentially regulated during *A. thaliana* defense (Widjaja et al., 2009). Even if storage proteins are not CWPs, they can be found as contaminants in specific cell wall proteomes, like seed cell wall proteomes (Merah et al., unpublished data). These depletion methods should then be useful to remove such major contaminants and improve the identification of low-abundant CWPs. Even more interesting and relevant for cell wall proteomics studies, is the new equalization technology based on the use of combinatorial hexapeptide ligand libraries (CPLLs) to reduce the dynamic range of protein concentrations (Fröhlich and Lindermayr, 2011). CPLLs consist in 64 millions different peptides fixed to a single bead commercially available. Specific binding of proteins depends on the physico-chemical properties of each protein. Highly-abundant proteins quickly saturate their ligands whereas all low-abundant ones are bound, resulting after elution, in a narrower dynamic range of all the proteins initially present. First successful results have been obtained for proteomics of spinach leaves (Fasoli et al., 2011), leaf extracts of *A. thaliana*, and phloem exudates of pumpkin (Fröhlich et al., 2012). Protein extraction is a critical step since native conformation of proteins is required for interaction with CPLLs. Notwithstanding the different limitations of this technique (Fröhlich and Lindermayr, 2011), it could be applied to the study of plant cell wall proteomes providing sufficient amounts of proteins are obtained. This approach would undoubtedly permit to identify new low-abundant proteins.

### Protein–protein interactions in cell walls

Many protein/protein interactions are expected in plant cell walls. Indeed, enzymes and their inhibitors like proteases and protease inhibitors, PMEs and PME inhibitors (PMEIs), or proteins with LRR domains have been detected in cell wall proteomes (Jamet et al., 2008). However, the present knowledge on plant CWPs suffers from the lack of data on protein–protein interaction mapping since most of the protein extraction methods used did not preserve supramolecular assemblies. One of the future challenges in plant cell wall proteomics will consist in developing extraction and capture methods to analyze CWP complexes. Concerning the purification of protein complexes, tandem affinity purification (TAP), in combination with MS, has become the method of choice to explore *in vivo* protein interactions (Xu et al., 2010). This method is based on the expression of a target protein fused to a double affinity tag. The first successful study of nuclear and cytoplasmic plant protein complexes using the TAP method has been carried out in a transient expression system of *N. benthamiana* (Rohila et al., 2004). The method has been optimized for use in plants. Since this first report, only a limited number of plant protein complexes through TAP have been reported from *A. thaliana* and *O. sativa* (Rohila et al., 2009; Andrès et al., 2011). It would be of special interest to carry out this method to analyze CWPs with predicted protein interaction domains, thus permitting to identify their partners. Further optimization will be necessary for (i) CWP extraction, possibly associated with protein cross-linking treatment, and (ii) protein complex capture which will require the design of a new TAP tag to preserve the level of accumulation of CWPs as well as their localization, stability, and function. Alternatively, the analysis of intact CWP assemblies could be conducted by applying low energy MS methods preserving non-covalent interactions developed in the frame of the analysis of mammalian or microbial protein complexes (Stengel et al., 2012). Such approaches should provide a more detailed description of plant CWP complex architecture.

### New MS tools to improve cell wall proteome description

Overcoming the future challenges in plant cell wall proteomics including analysis of low-abundance proteins, PTMs, protein–protein interactions, and quantitative proteomics will be facilitated by significant advances in MS technologies (Thelen and Miernyk, 2012). MS instrumentation evolves very quickly and impressive improvement in sensitivity, mass accuracy and fragmentation has been achieved in recent years. Instruments like Fourier Transformed-Ion Cyclotron Resonance (FT-ICR) are capable of mass accuracy of less than 2 ppm and have a high resolution (above 10^6^). Sensitivity of new generation MS instruments reaches the femtomole or the attomole range. New fragmentation methods such as electron capture dissociation (ECD) and electron transfer dissociation (ETD) (Bond and Kohler, 2007) are also very promising. They will provide new insight into the structure of CWPs, as recently achieved for the AGP31, an *A. thaliana* cell wall *O*-glycoprotein (Hijazi et al., 2012). Finally, progresses in bioinformatics will be very helpful to characterize cell wall glycoproteins. Several computer programs like GlycoMod (Cooper et al., 2001), GlysodeIQ^TM^(Joshi et al., 2004), GlycoMiner (Ozohanics et al., 2008), or Peptoonist (Goldberg et al., 2007) have been developed, but most of them do not consider plant glycan specificities. The *ProTerNyc* software has been developed in this purpose and efficiently used to predict *N*-glycan motifs on cell wall glycoproteins (Albenne et al., 2009; Zhang et al., 2011). However, additional bioinformatics tools should be developed to improve automatic data interpretation.

## Beyond cell wall proteomics

### Identification of candidate proteins and search for function

In addition to the basic work of protein identification resulting in lists of proteins, cell wall proteomics has become a new tool to identify candidate proteins involved in developmental processes or in response to environmental cues. Some examples involve quantitative analyses. Up to now, label-free techniques have been favored like quantification of stained spots on polyacrylamide gels (Ndimba et al., 2003; Oh et al., 2005; Tran and Plaxton, 2008), spectral counting (Irshad et al., 2008) or calculation of area under the curve (AUC) (Cheng et al., 2009). Only one study has been performed with the difference in gel electrophoresis (DIGE) technique which requires the labeling of proteins with fluorescent dyes prior to electrophoresis (Casasoli et al., 2008). Quantifications performed on stained spots are difficult to interprete since some proteins are present in different spots for different reasons such as presence of PTMs, degradation or maturation of proteins. In addition, contrary to staining with fluorescent molecules like Sypro® Ruby, staining with Coomassie blue or silver nitrate has a narrow dynamic range, i.e., about two orders of magnitude (Moritz and Meyer, 2003). Only a few of these proteomics studies has given rise to functional or structural studies of proteins. A protein containing a GDSL motif lipase/hydrolase (GLIP1) has been identified as one of the salicylic acid (SA) responsive proteins secreted by *A. thaliana* cell suspension cultures (Oh et al., 2005). The increase in protein level was calculated to be three-fold after comparison of proteins extracted from control and from SA-treated cells separated by 2D-E and stained with silver nitrate. Two *glip1* T-DNA insertion mutants have been found to be more resistant to the *Alternaria brassicicola* necrotrophic fungus. It has also been shown that the recombinant GLIP1 protein has a lipase activity and an antimicrobial activity able to disrupt the integrity of fungal spores. The AGP AGP31 has been identified as a major protein in the cell wall proteome of etiolated hypocotyls of *A. thaliana* (Irshad et al., 2008). AGP31 is a multi-domain proteins having a N-terminal AGP, a central Pro-rich and a C-terminal Cys-rich domains. The combination of several MS technologies has allowed the first description of the Pro hydroxylation and *O*-glycosylation patterns of its Pro-rich domain (Hijazi et al., 2012). Finally, the *N. tabacum* NtSCP1 serine carboxypeptidase III identified in leaf intercellular fluids has been later shown to be involved in cell elongation (Delannoy et al., 2008; Bienert et al., 2012). The protease activity of NtSCP1 has been demonstrated *in vitro* and its cell wall localization has been confirmed by expression of the protein as a GFP fusion protein *in vivo*. Over-expression of *NtSCP1* has led to reduce flower length due to decrease in cell size and to etiolated seedlings with short hypocotyls.

In addition to proteomics data, it would be interesting to consider other data to identify proteins of interest such as transcriptomics or gene regulatory networks. Such data are available online (e.g., http://bar.utoronto.ca/welcome.htm, https://www.genevestigator.com/gv/, http://aranet.mpimp-golm.mpg.de/, http://atted.jp/). The feeding of new portals like MASCP Gator which aims at unifying the *A. thaliana* proteomics resources in a single interface for the research community is also essential (Joshi et al., 2011). This systems biology approach would allow a better understanding of gene regulation, from gene transcription to protein synthesis and even PTMs, allowing the description of protein active forms. Indeed, several studies have shown that proteomics and transcriptomics data are complementary and do not give exactly the same picture of a physiological situation depending on the level of regulation of gene expression (Jamet et al., 2009; Minic et al., 2009).

### From proteomics to peptidomics

During the last decade, it has become evident that secreted peptides function as signaling molecules in cell-to-cell communication in plants. Recently, they have been recognized as hormones that coordinate and specify cellular functions in complex developmental processes (Shinohara and Matsubayashi, 2010; Murphy et al., 2012). The secreted signaling peptides identified so far can be categorized in two groups: (i) the small post-translationally modified peptides are less than 20 amino acids and undergo extensive proteolytic processing from a longer precursor and PTMs such as Tyr sulfation, Pro hydroxylation and arabinosylation on Hyp; (ii) the Cys-rich peptides are larger (<160 amino acids), cationic at the extracellular pH and have multiple intra-molecular disulfide bonds. All of them have a predicted signal peptide. Peptidomics is becoming a stimulating field especially because of the description of the active forms of the signaling peptides (Shinohara and Matsubayashi, 2010; Murphy et al., 2012). Indeed, the PTMs are essential for their biological activity. For instance, STOMAGEN, a Cys-rich peptide that positively regulates stomatal density in *A. thaliana*, is active at nanomolar (10 nM) concentrations when forming three disulphide bonds. When Cys residues were replaced by Ser residues, STOMAGEN was unable to increase stomatal density even at very high (10 μM) concentrations (Ohki et al., 2011). Tyr sulfation and arabinosylation were also required for the full activity of small post-translationally modified peptides (Ohyama et al., 2009; Matsuzaki et al., 2010). Like cell wall proteomics, peptidomics has the potential to reveal new secreted signaling peptides as well as new functions of plant cell walls.

Today, the description of cell wall peptidomes, or secreted peptidomes, defined as a set of peptides present in cell walls at a specified physiological state, is lacking. Two main reasons can explain such a gap. First, the signaling peptides are believed to be present in very low quantity in plant tissues, they are active at nanomolar concentrations and their transcripts have been found to be transiently expressed (Ito et al., 2006; Chen et al., 2008b). Most of the well-characterized signaling peptides have been identified by genetics and *in silico* approaches (Murphy et al., 2012). In order to fully characterize the structure of their mature forms, they have been produced by cells or plants over-expressing the corresponding genes to obtain sufficient amounts of peptides amenable to LC-MS-based structure analysis (Amano et al., 2007; Ohyama et al., 2008, 2009; Sugano et al., 2010) or to *in situ* MALDI-TOF-MS analysis (Kondo et al., 2006). The latter study used *A. thaliana* plants over-expressing *CLV3*, a gene encoding a 96 amino acid propeptide containing a signal peptide. *CLV3* has been shown to be involved in the control of the size of the shoot apical meristem. The identified mature peptide contained 12 amino acids from Arg^70^ to His^81^ in CLV3 in which two of three Pro residues were modified to Hyp. Nevertheless, a number of studies that employed LC purification and Edman sequencing or MS identification have been developed and applied successfully to the analysis of native peptide sources (Pearce et al., 1991; Matsubayashi and Sakagami, 1996; Ito et al., 2006; Chen et al., 2008b). When these studies used high amounts of plants as starting material, they have allowed the identification of mature signaling peptides active at a concentration of 10^−11^M in the case of TDIF (Tracheary element Differentiation Inhibitory Factor) (Ito et al., 2006). The corresponding cDNA of *Zinnia elegans* encodes a protein of 132 amino acids, but only 12, from His^120^ to Asn^131^, match the TDIF sequence with two Hyp residues (Hyp^123^ and Hyp^126^).

Second, peptide-encoding genes are frequently overlooked during the annotation of genomes. Indeed, gene prediction programs hardly distinguish between short, often intronless peptide-encoding genes and random small open reading frames (ORFs). To minimize incorrect gene predictions, it is common that small ORFs are rejected (Olsen et al., 2002). To overcome such a deficiency, a bioinformatics approach has been undertaken to identify candidate peptide-encoding genes in the *A. thaliana* genome (Lease and Walker, 2006). It has led to an unannotated secreted peptide database containing 33,809 ORFs. The identified peptides have been characterized by the presence of a predicted N-terminal signal peptide and by the absence of transmembrane domains and ER retention sequences. Since the expression of some ORFs has been detected by RT-PCR, it is suggested that the number and diversity of plant peptides is broader than currently assumed (Lease and Walker, 2006). The secreted peptide database will permit the necessary retrieval of information required for the identification of *A. thaliana* signaling peptides. Together with the progress of MS sensitivity, cell wall peptidomics is now a reachable objective.

## Conclusion: up and coming of cell wall proteomics

Within the last 10 years, cell wall proteomics studies have received full credit among the OMICS strategies. They have allowed not only the precise identification of proteins in particular physiological conditions, but also their quantification and the characterization of their PTMs. Proteomics could also provide information about the dynamics of CWPs by kinetics analysis to follow the *de novo* synthesis of proteins or their degradation during plant development or in response to environmental cues. All the knowledge presently available on cell wall proteomics contributes to a better understanding of CWP structures and functions in cell walls. However, it is not yet possible to distinguish proteomes of primary and secondary walls notably because it is difficult to separate the cells surrounded by either of them. Micro-dissection of tissues should help solving this problem providing enough material can be obtained, but the extraction of proteins from the intricate macromolecular networks of secondary walls remains a great challenge. Next development will take advantage of cutting-edge MS technologies for a better coverage of cell wall proteomes, a more precise description of protein forms and protein complexes and for an insight into cell wall peptidomics.

### Conflict of interest statement

The authors declare that the research was conducted in the absence of any commercial or financial relationships that could be construed as a potential conflict of interest.

## Appendix

**Table A1:** **Cell wall proteomes and secretomes of *A. thaliana*: detailed information**.

**Organ**	**References**	**Destructive or non-destructive method**	**Type of proteome**	**Buffer for protein extraction**	**Protein separation**	**Protein identification**	**Total number of identified proteins**	**Number of proteins with predicted target signal and no predicted intracellular retention signal**
Step (see Figure 1)		Step 1		Step 2	Step 3	Step 4		
Culture medium of roots	Basu et al. (2006)		Secretome			2D-LC-MS/MS	52	36
Stems	Minic et al. (2007)	Destructive (grinding)	N-glycoproteome (ConA affinity chromatography)	Salt solution (200 mM CaCl2)	2D-E	LC-MS/MS	102	90
Leaves	Haslam et al. (2003)	Non-destructive (vacuum infiltration)	Apoplastic proteome	low ionic strength buffer	2D-E	MALDI-TOF MS	10	9
Leaves	Boudart et al. (2005)	Non-destructive (vacuum infiltration)	Apoplastic proteome	0.3 M mannitol or salt solutions (1 M NaCl, 200 mM CaCl2, 2 M LiCl, 50 mM CDTA), acidic pH	1D-E or 2D-E	MALDI-TOF MS	93	87
Etiolated hypocotyls (5 days)	Feiz et al. (2006)	Destructive (grinding in low ionic strength acidic buffer), cell wall purification	Cell wall proteome	Salt solutions (200 mM CaCl2, 2 M LiCl), acidic pH	1D-E	MALDI-TOF MS	96	84
Etiolated hypocotyls (5 and 11 days)	Irshad et al. (2008)	Destructive (grinding in low ionic strength acidic buffer), cell wall purification	Cell wall proteome	Salt solutions (200 mM CaCl2, 2 M LiCl), acidic pH	Cationic exchange chromatography followed by 1D-E	MALDI-TOF MS	173	137
Etiolated hypocotyls (11 days)	Zhang et al. (2011)	Destructive (grinding in low ionic strength acidic buffer), cell wall purification	Cell wall glycoproteome (lectin affinity chromatography, boronic acid chromatography)	Salt solutions (200 mM CaCl2, 2 M LiCl), acidic pH	1D-E	MALDI-TOF MS and LC-MS/MS	129	127
Seedlings (10 days) (oligogalacturonides)	Casasoli et al. (2008)	Non-destructive	Apoplastic proteome	Salt solution (150 mM MgCl2, 50 mM EDTA), basic pH	2D-E	MALDI-TOF MS and LC-MS/MS	20	14
Cell suspension cultures (3 days)	Bayer et al. (2006)	Destructive (grinding in salt solution)	Cell wall proteome	Phenol buffer		2D-LC-MS/MS	792	106
Cell suspension cultures (5 days)	Kwon et al. (2005)	Non-destructive (washing of cells)	Cell wall proteome	Salt solution (1 M KCl)	2D-E	MALDI-TOF MS	39	34
Cell suspension cultures (5 days)	Chivasa et al. (2002)	Destructive (grinding)	Cell wall proteome	Salt solution, chaotropic agent (urea and thiourea) and detergent (CHAPS)	2D-E	MALDI-TOF MS	72	26
Cell suspension cultures (5 days)	Robertson et al. (1997)	Non-destructive (washing of cells)	Cell wall proteome	Salt solutions (0.2 M CaCl2, 50 mM CDTA, 1 M NaCl, 0.2 M borate)	1D-E	Edman N-terminal sequencing	33	18
Cell suspension cultures (7 days)	Borderies et al. (2003)	Non-destructive (washing of cells)	Cell wall proteome	Glycerol solution (50%) or salt solutions (successively 0.15 M NaCl, 1 M NaCl, 50 mM EDTA, 2 M LiCl in 50% glycerol)	1D-E or 2D-E	MALDI-TOF MS	95	50
Culture medium of calli (21 days	Borner et al. (2003)	Destructive (purification of a membrane fraction with detergent)	GPI-anchored proteome	Pi-PLC treatment, detergent (Triton X-114)	1D-E or 2D-E	LC-MS/MS	30	30
Culture medium of cell suspension cultures (4 days)	Oh et al. (2005)		Secretome		2D-E	MALDI-TOF MS	45	24
Cell suspension cultures (7 days) (fungal elicitors)	Ndimba et al. (2003)	Destructive (grinding)	Cell wall proteome	Salt solution (200 mM CaCl2)	2D-E	MALDI-TOF MS	6	6
Culture medium of cell suspension cultures (7 days) (salicylic acid treatment)	Cheng et al. (2009)		Secretome			2D-LC-MS/MS	74	39
Culture medium of cell suspension cultures (7 days) (phosphate deficiency)	Tran and Plaxton (2008)		Secretome		2D-E	MALDI-TOF MS	37	21
Protoplasts (1 h)	Kwon et al. (2005)	Non-destructive (washing of cells)	Cell wall proteome	Salt solution (1 M KCl)	2D-E	MALDI-TOF MS	71	25
Protoplasts (3 h)	Kwon et al. (2005)	Non-destructive (washing of cells)	Cell wall proteome	Salt solution (1 M KCl)	2D-E	MALDI-TOF MS	66	23
Liquid cultured etiolated seedlings	Schultz et al. (2004)	Destructive (grinding)	Arabinogalactan proteins (AGPs) proteome	Detergent-containing buffer (Triton X-100), Yariv precipitation, HF deglycosylation	HPLC	Edman N-terminal sequencing	12	12
Culture medium of etiolated seedlings	Charmont et al. (2005)		Secretome		2D-E	MALDI-TOF MS	49	44
